# Pathological Findings in Gastrointestinal Neoplasms and Polyps in 860 Cats and a Pilot Study on miRNA Analyses

**DOI:** 10.3390/vetsci9090477

**Published:** 2022-09-03

**Authors:** Alexandra Kehl, Katrin Törner, Annemarie Jordan, Mareike Lorenz, Ulrike Schwittlick, David Conrad, Katja Steiger, Benjamin Schusser, Heike Aupperle-Lellbach

**Affiliations:** 1 LABOKLIN GmbH & Co. KG, 97688 Bad Kissingen, Germany; 2 Comparative Experimental Pathology, School of Medicine, Technical University of Munich (TUM), 81675 Munich, Germany; 3 Reproductive Biotechnology, Department of Molecular Life Sciences, School of Life Sciences Weihenstephan, Technical University of Munich, 85354 Freising, Germany

**Keywords:** lymphoma, carcinoma, sarcoma, polyp, mast cell tumour, intestine, stomach, histology, immunohistochemistry, PCR

## Abstract

**Simple Summary:**

In cats, gastrointestinal masses appear in relevant numbers. Histopathology and immunohistochemistry are needed for a detailed diagnosis but require invasive sampling. Therefore, the improvement of presurgical diagnostics (e.g., biomarkers in serum) is of interest in feline medicine. The present analysis of pathology reports included 679 alimentary lymphomas, 122 carcinomas, 29 spindle cell tumours, 23 polyps and 7 mast cell tumours (MCT). Immunohistochemical characterisation was available from 91 lymphomas, 10 sarcomas and 7 MCTs. Carcinomas and polyps were most commonly found in the large intestine, lymphomas were most commonly found in the stomach and small intestine and MCTs only occurred in the small intestine. In 68%, the submitted lymph nodes were infiltrated by neoplasms, and surgical margins were often not free of tumour cells. The prognostic and therapeutic value of cell size, mitotic count and immunophenotype in lymphomas must be interpreted carefully. A pilot study on miRNA-20b and miRNA-192 concentration in 11 lymphomas, 5 carcinomas and 5 controls was performed. The values of miRNA-20b were found to be up-regulated in samples of all types of cancer, whereas miRNA-192 was up-regulated in carcinomas and B-cell lymphomas only. The diagnostic purpose of miRNAs as potential biomarkers used for the non-invasive diagnosis of intestinal cancer in cats should be further evaluated.

**Abstract:**

Background: Gastrointestinal masses in cats are of clinical relevance, but pathological studies with larger case numbers are lacking. Biomarkers such as miRNA have not yet been investigated in feline intestinal neoplasms. Methods: A retrospective analysis of pathology reports included 860 feline gastrointestinal masses. Immunohistochemistry was performed on 91 lymphomas, 10 sarcomas and 7 mast cell tumours (MCT). Analyses of miRNA-20b and miRNA-192 were performed on 11 lymphomas, 5 carcinomas and 5 control tissues by ddPCR. Results: The pathological diagnosis identified 679 lymphomas, 122 carcinomas, 28 sarcomas, 23 polyps, 7 MCT and 1 leiomyoma. Carcinomas and polyps were most commonly found in the large intestine, lymphomas were most commonly found in the stomach and small intestine and MCT only occurred in the small intestine. Besides the well-described small-cell, mitotic count <2 T-cell lymphomas and the large-cell B-cell lymphomas with a high mitotic count, several variants of lymphomas were identified. The values of miRNA-20b were found to be up-regulated in samples of all types of cancer, whereas miRNA-192 was only up-regulated in carcinomas and B-cell lymphomas. Conclusions: The histopathological and immunohistochemical (sub-)classification of feline intestinal masses confirmed the occurrence of different tumour types, with lymphoma being the most frequent neoplasm. Novel biomarkers such as miRNA-20b and miRNA-192 might have diagnostic potential in feline intestinal neoplasms and should be further investigated.

## 1. Introduction

Nodular or diffuse proliferations in the gastrointestinal tract of cats may be associated with non-specific gastrointestinal signs such as inappetence, vomiting and emaciation [1]. Imaging studies provide further evidence of the exact location, the organ of origin, and the extent of intra-abdominal masses [2,3]. Cytology is often of limited diagnostic value in those cases. Therefore, biopsy sampling or the resection of the complete mass with subsequent histological examination is usually essential for a detailed diagnostic workup [1,4]. In addition to inflammatory changes [5] and tumour-like lesions such as polyps [6], various tumours [7] have been described in the gastrointestinal tract of cats. The identification of one or more potential molecular biomarkers [8,9,10] as part of non-invasive diagnostics in serum (”liquid biopsy”) could contribute decisively to the refinement of diagnostic methods in companion animals [11].

In recent years, microRNAs (miRNAs) have gained greater attention, as they seem to be able to serve as biomarkers for diagnosing and monitoring [12]. MicroRNAs are small non-coding RNAs which regulate the expression of genes by translational suppression or enhancement [13]. There is strong evidence that miRNAs play a role in oncogenesis by having tumour-suppressive or oncogenic effects [14]. To clarify the functions of miRNAs in the development of cancer, the miRNA profiles of healthy tissue and cancer tissue must be compared. However, expression shifts can vary significantly between different types of cancer. Only few publications are available on miRNA profiles in feline neoplasms [15,16,17], and, to the best of our knowledge, no studies on miRNA expression in feline intestinal neoplasms have been conducted yet. In dogs, the miRNA profile of intestinal T-cell lymphoma was determined, with the expression of miRNA (miR)-192, 194, 141 and 203 being down-regulated and the expression of miR-20, 18b and 363 being up-regulated [18]. 

Lymphomas are known to be the most common intestinal neoplasm in cats [7,19]. However, Avallone et al. recently concluded that specific guidelines for the histological grading of animal lymphomas located in anatomical sites other than the lymph nodes (e.g., alimentary tract, respiratory tract, skin) have not yet been established [20]. The veterinary World Health Organisation (WHO) nomenclature classifies alimentary lymphomas according to the size of the neoplastic cells and the immunophenotype as: (1) extra-nodal marginal zone B-cell lymphoma of the mucosa-associated lymphatic tissue (MALT), (2) intestinal T-cell lymphoma (small-cell), (3) T-cell large granular lymphoma (LGL) and (4) multicentric lymphomas which do not affect the gastrointestinal tract alone. Furthermore, a modified nomenclature according to human medicine was suggested [21,22]: (1) diffuse large B-cell lymphoma (DLBCL), (2) mucosal lymphoma of the MALT, (3) enteropathy-associated T-cell lymphoma (EATL) with type 1: large-cell with necrosis and inflammation and type 2: small- to medium-sized without necrosis and inflammation and (4) T-cell large granular lymphoma (LGL). However, this translation from the human system to feline alimentary lymphomas has well-known limitations [23]. Moreover, it was described that LGL can normally only be diagnosed by cytology because granules are not visible in most histological cases [24]. 

The histomorphological criteria of alimentary lymphomas vary considerably in the literature, and no grading system with prognostic relevance has been established so far [20]. In intestinal lymphomas 0–2 mitotic figures/high-power fields (hpf) have been described [25]. In contrast, lymphomas of the lymph nodes were classified as low-grade if they had fewer than 5 mitotic figures/hpf, medium-grade if they had between 5 and 10 and high-grade if they had over 10 mitotic figures/hpf [26]. A valid, clinically controlled grading study defining the number of mitoses for medium- and high-grade alimentary lymphomas is not available. Immunophenotyping is relevant for the prognosis and treatment of alimentary lymphoma [1]. It is well known that, in general, immunohistochemistry is the gold standard for immunophenotyping into T-cell, B-cell or T-cell-rich B-cell lymphomas from any anatomical site. In cases of doubt (e.g., follicular lymphomas), clonality analysis (PARR) may be useful, but, especially in cats, this method has several limitations, as described by various authors [19,27,28]. 

Adenocarcinomas are the second most common intestinal neoplasm in cats [7]. Morphological and molecular genetic findings in feline intestinal carcinomas have been described in detail by Groll and colleagues [29]. Furthermore, mast cell tumours [30,31,32,33] and sarcomas [34,35,36,37,38] have been reported in the feline gastrointestinal tract. 

A retrospective study of 1129 feline intestinal neoplasms [7] mainly focused on epidemiological analyses including age, gender and breed. However, there are currently no pathology-based studies with high case numbers of feline intestinal tumours (especially lymphomas) and polyps available. The aims of this study were: (1) to retrospectively describe the frequency, histomorphology and immunohistochemical characteristics of feline intestinal neoplasms submitted for routine diagnosis, with a special view on lymphomas; (2) to report the results of a pilot project for establishing a method of miRNA analysis in feline intestinal neoplasms.

## 2. Materials and Methods

### 2.1. Histopathology

The datasets were derived from 85,887 cats (January 2013–April 2022). They were obtained from the histopathological diagnostic service from LABOKLIN GmbH & Co. KG, Bad Kissingen, Germany, and analysed retrospectively. The data were extracted from the proprietary database into an Excel file and included breed, age, sex, gross pathology, histological findings, diagnosis and epicritic comments as well as additional results of immunohistochemistry, as far as available. Information on breed, age, sex and sample site were taken from the submission forms. They had to be assumed to be accurate and were standardised for statistical analysis.

The inclusion criteria were: (1) only histopathological samples (no cytological or necropsy cases) were evaluated; (2) the breed, age and sex of the cat were known; (3) the gastric or intestinal origin of the neoplasm—especially in the case of carcinomas—could be clearly identified; (4) a definitive diagnosis was possible. Although polyps are classified by WHO classification as a non-neoplastic tumour-like lesion [39], polyps were included in the present study because they are an important (benign) differential diagnosis in cases with gastrointestinal masses [6,40,41].

Finally, 860 cases fulfilled all criteria and were included in this study. These were mucosal and transmural biopsies as well as resected segments from the stomach and small and/or large intestine. In 307 cases, additional tissue of regional lymph nodes was submitted. 

The preparation of formalin-fixed tissue samples including the macroscopic description (e.g., size of the mass) was performed according to trimming guidelines [42]. Samples were embedded in paraffin wax and cut into 2–3 µm-thick slices which were stained with haematoxylin-eosin. Where necessary, special staining (e.g., Giemsa, toluidine blue) was performed according to standard protocols [43]. The diagnoses were routinely made by means of light microscopic examination and were reported by veterinary pathologists of varying qualifications within the diagnostic team. 

Although there is no established grading system recommended for feline alimentary lymphoma, cell size and mitotic count seem to be relevant criteria [20]. Thus, we reported the size of the neoplastic lymphocyte population as small-cell, intermediate-sized and large-cell lymphoma, as defined by the WHO [44]. In general, small-cell lymphomas were characterised by small, round neoplastic cells (1–1.5× the size of a red blood cell) with variably defined cell borders, a scant amount of slightly eosinophilic cytoplasm (N:C ratio 1:1) and a single round basophilic nucleus stippled to the vesicular chromatin. Medium-sized (1.5–2× the size of a red blood cell) or large (>2× the size of a red blood cell) neoplastic lymphocytic cells had variably defined cell borders, a scant to moderate amount of slightly eosinophilic cytoplasm and one round nucleus with a single nucleolus or multiple prominent nucleoli.

Intestinal carcinomas were classified as tubular (stomach) or acinar (intestine), papillary, undifferentiated (solid), signet-ring type, (adeno)squamous or mucinous [29,39] if most parts (approximately 75%) of the neoplastic tissue showed a morphology compatible with one pattern or the other. 

Mast cell tumours were classified as well, moderately or poorly differentiated, as previously described [30]. A special form with increased amounts of fibrous tissue was referred to as a sclerosing mast cell tumour [31].

In general, the mitotic count was evaluated in 10 hpf (FN 22/40x, area: 2.37 mm², Nikon Eclipse E200 microscope; Nikon, Tokyo, Japan) in the areas with the highest mitotic activity [45]. In lymphomas, carcinomas and mast cell tumours, the average mitotic count/hpf—and, in sarcomas, the sum of mitotic counts (mitoses/10 hpf)—was calculated. 

### 2.2. Immunohistochemistry

Immunohistochemical examination was performed on 91 lymphomas (CD3, CD20, CD79a), 10 sarcomas (cKit/CD117, smooth muscle alpha actin, glial fibrillary protein) and 7 mast cell tumours (cKit/CD117) at the request of the clients.

Immunohistochemistry was conducted according to a standard protocol. Briefly, tissue sections were mounted on coated slides (SuperFrost^®^ Plus, Menzel Gläser, Thermo Fisher Scientific, Waltham, MA, USA). Pre-treatment for antigen-demasking (Table 1) was performed at a high temperature (96 °C) with EDTA buffer (pH 9.0, HIER T-EDTA pH 9.0, Zytomed #ZUC029-500, Zytomed System GmbH, Berlin, Germany) in a conventional steam heater for 25 min or with a target retrieval solution buffer (Dako, #S1699; Dako Denmark A/S, Glostrup, Denmark) in a conventional pressure cooker for 10 min, respectively. 

Primary monoclonal antibodies (Table 1), known to be cross-reacting with feline tissue, were diluted in antibody diluent (Zytomed, #ZUC025-100, Zytomed System GmbH, Berlin, Germany) and applied on cover plates (Shandon Coverplate^TM^, Thermo Scientific #A73310024, Thermo Fisher Scientific, Cheshire, UK). Feline lymph node, smooth muscle and nervous tissue served as positive controls. Sections were incubated with the primary antibodies at room temperature for 60 min. For negative controls, the primary antibody was replaced by a buffer. A commercial detection system, the ZytoChem Plus HRP Polymer Kit (#POLHRP-100, Zytomed System GmbH, Berlin, Germany), was applied for 30 min at room temperature. All slides were finally incubated with a chromogen (DAB (diaminobenzidine tetrahydrochloride), Dako, #K3468; Dako Denmark A/S, Glostrup, Denmark) for 10 min at room temperature and counterstained with hemalum. The evaluation of the immunohistochemical cases, including the controls, was performed by one pathologist of the diagnostic team.

### 2.3. miRNA Analysis

A molecular genetic analysis of miRNA expression was performed in randomly chosen large-cell B-cell lymphomas (*n* = 6), small-cell T-cell lymphomas (*n* = 5) and tubular carcinomas (*n* = 5) of the small intestine. Five samples of normal small intestinal tissue from routine diagnostics without inflammatory or neoplastic changes (derived from the archive described in 2.1.) served as controls (Table 2)**.**

The total miRNA was isolated from two 10 µm-thick tissue paraffin sections (not mounted on a slide) using the miRNeasy FFPE Kit (QIAGEN, #217504, Hilden, Germany), according to the manufacturer’s instructions. The total miRNA was eluted in 40 µL of RNase-free water and diluted 1:5 with RNase-free water. Expression analysis was conducted by droplet digital PCR (ddPCR) using specific microRNA assays (Thermo Fisher Scientific, #4427975, Waltham, MA, USA). Reverse transcription was performed using the TaqManTM MicroRNA Reverse Transcription Kit (Thermo Fisher Scientific, #4366597, Waltham, MA, USA), according to the manufacturer’s instructions, with the specific primer from the TaqManTM miRNA assay (Thermo Fisher Scientific, #4427975, Waltham, MA, USA). cDNA was diluted 1:10 with RNase-free water.

The ddPCR was run on the QX200 Droplet Digital System (Bio-Rad, Hercules, CA, USA) using ddPCR supermix for probes (Bio-Rad, Hercules, CA, USA) and a specific TaqMan^TM^ miRNA assay (Thermo Fisher Scientific, #4427975, Waltham, MA, USA). All miRNAs that were tested in intestinal T-cell lymphoma in dogs [18] were screened for the availability of a cat-specific, commercially available miRNA assay. TaqMan^TM^ miRNA assays were tested for their functionality in ddPCR. Finally, miR-20b (assay ID 001014) and miR-192 (assay ID 000493) were chosen as targets, reflecting one up-regulated (probably oncogenic) and one down-regulated (probably tumour-suppressive) miRNA. Additionally, the RNU6B (assay ID 001973) value was determined and used as a normaliser. A total of 10 µL cDNA was added to an 11 µL ddPCR supermix and 1 µL TaqMan^TM^ miRNA assay. Droplets were generated using the Droplet Maker (Bio-Rad, Hercules, CA, USA). PCR was run with 35 cycles of 94 °C for 30 s, 60 °C for 30 s and 72 °C for 30 s. The measurement was performed using the QX200 Droplet Reader and QuantaSoft software (Bio-Rad, Hercules, CA, USA). Each PCR was performed in triplicate. The validity of our test system was checked by inter- and intra-assay tests as being robust, with a variation coefficient of less than 0.15. The triplicate data of the samples described herein showed a high reproducibility, with a variation coefficient of 0.11 for miR-192 and of 0.13 for miR-20b.

### 2.4. Statistics

The statistical analysis of all of the included cases was performed with IBM SPSS statistics for Windows (version 28.01.1, IBM, Armonk, NY, USA). The Kolmogorov–Smirnov and Shapiro–Wilk tests were used to test the data for normal distribution. The *p*-values were adjusted for multiple testing using the Bonferroni correction. Comparison between the groups was carried out using the Kruskal–Wallis test or the Mann–Whitney–U test. It was considered statistically significant if the *p*-values were < 0.05 (* < 0.05; ** < 0.01; *** < 0.001).

## 3. Results

### 3.1. Total Cat Population with Gastrointestinal Non-Inflammatory Masses (n = 860)

In addition to 604 domestic short-haired cats (DSH), the most common breeds in this study were mixed breeds (*n* = 77), British shorthair (BSH, *n* = 39), Maine coon (*n* = 30), Persian (*n* = 17), Norwegian forest cat (*n* = 15), Siamese (*n* = 10) and Chartreux (*n* = 9). The other 17 breeds were included, with a few cats each. The age of the cats ranged from 1 to 22 years (median: 11 years; 25% quartile: 8 years; 75% quartile: 13 years). Most neoplasms (64.7%) were found in cats older than ten years, and 3% appeared in cats younger than 4 years. There were 73 intact and 308 spayed females as well as 75 intact and 404 castrated male cats.

**Figure 1:**
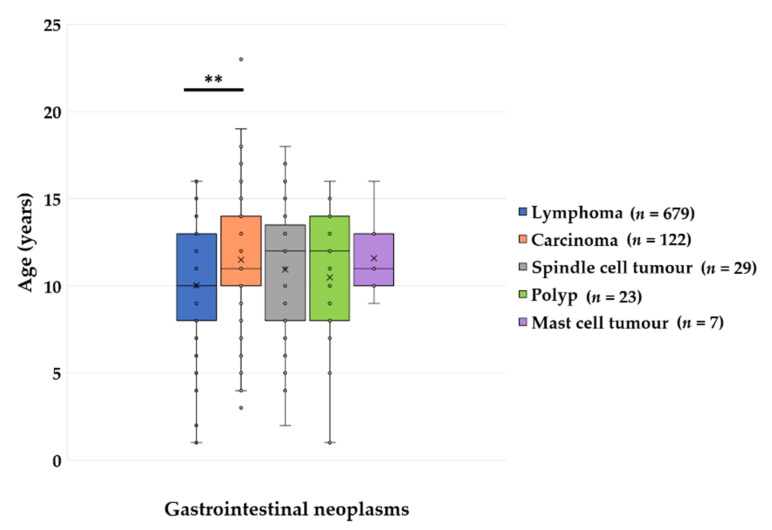
The age of the cats with different gastrointestinal neoplasms and polyps showed a wide variation. Cats with alimentary lymphomas were significantly younger than cats with carcinomas (**, *p* = 0.005).

Most gastrointestinal proliferations found in these 860 cats were malignant (97.3%), with the exception of 23 polyps (2.7%) and 1 leiomyoma. Lymphomas (*n* = 679; 81.2%) were the most common malignant gastrointestinal tumours, followed by 122 carcinomas (14.6%), 28 sarcomas (3.4%), and 7 mast cell tumours (0.8%). In general, cats with lymphomas were significantly younger than cats with carcinomas (*p* = 0.005, Figure 1).

**Figure 2:**
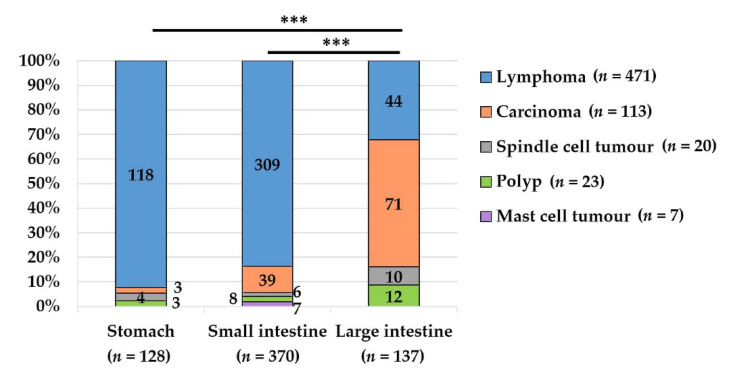
The frequency of different gastrointestinal neoplasms varies depending on the anatomical site. The differences between the stomach and large intestine (***, *p* < 0.001), as well as between the small intestine and large intestine (***, *p* < 0.001), were significant. Carcinomas occurred more frequently in the large intestine than in the small intestine or the stomach (intestine NOS and lymphomas in more than one sample are not included in the graph).

The histopathological diagnoses varied significantly between the large intestine and the stomach (*p* < 0.001), as well as between the large and the small intestine (*p* < 0.001) (Figure 2). Histopathologically diagnosed carcinomas were more frequent in the large intestine, while lymphomas were mainly located in the stomach and the small intestine, and mast cell tumours occurred exclusively in the small intestine. Due to the destructive neoplastic growth in smaller samples and the missing clinical information, it was not always possible to identify the correct intestinal site by histology. These cases were termed “intestine not otherwise specified” (intestine NOS). Neoplasms were mostly (82.1%) located in the intestine (small intestine *n* = 370, large intestine *n* = 137, intestine NOS *n* = 199). A total of 128 were located in the stomach (14.9%), and, in 26 cases (3%), neoplasms were identified in gastric and intestinal samples from the same animal.

### 3.2. Lymphoma, n = 679

The age was not significantly correlated with the gastrointestinal site of the mass. The median age of the cats was 10 years (25% quartile: 8 years; 75% quartile: 13 years). Cats suffering from alimentary lymphomas were significantly younger than cats with carcinomas (*p* = 0.005, Figure 1). However, 62.2% of the animals with lymphomas were ≥10 years old. There were 54 intact and 250 spayed females, as well as 60 intact and 315 castrated male cats. In addition to 477 DSH, the most common breeds in this group were mixed breeds (*n* = 58), BSH (*n* = 28), Maine coon (*n* = 25), Persian (*n* = 12), Norwegian forest cat (*n* = 12), Siamese (*n* = 9), Bengal (*n* = 6) and Russian blue (*n* = 6) (Table 3).

**Figure 3:**
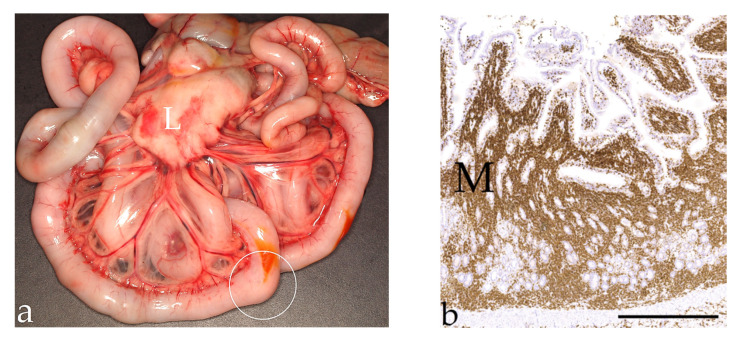
Alimentary lymphoma. (**a**): Macroscopy of a markedly enlarged lymph node (L) and a nodular mass (circle) in the small intestine of a 13-year-old male domestic short-haired cat. (**b**): Immunohistochemistry of a T-cell lymphoma (small cell) with intense CD3 expression of neoplastic lymphocytes visible in the mucosa (M) of a 12-year-old male castrated DSH; (IHC, bar = 600 µm).

Alimentary lymphomas were most often (78.9%) located in the intestine (small intestine *n* = 310, large intestine *n* = 44, intestine NOS *n* = 182), but 17.4% were located in the stomach, and 3.7% of the neoplasms were identified in both gastric and intestinal samples. In 131 cases, gastrointestinal segments with complete nodular masses were submitted (Figure 3a). The size of the masses was independent of where they were located in the gastrointestinal tract. The size of the completely submitted masses (*n* = 92) ranged from 0.9 × 0.5 × 0.3 cm to 8.0 × 8.0 × 4.3 cm. The other lymphomas were diagnosed in biopsies, and no (clinical) information about the neoplastic size was available.

Microscopically, the gastric and/or intestinal mucosa (*n* = 49), mucosa and lamina propria (*n* = 24) and mucosa, lamina propria, tunica muscularis and serosa (*n* = 606) were diffusely and markedly infiltrated by a lymphocytic tumour cell population. However, it should be noticed that not all samples were transmural samples, and further extension to the muscularis could not be excluded in 37 cases (especially from the stomach). The regional lymph node was affected in 170 of 258 biopsied cases (69.7%), regardless of the gastrointestinal location of the neoplasm or the morphology of the neoplastic cells. Lymph nodes were involved in 155 out of 232 transmural lymphomas (66.8%) and in 15 out of 26 cases (57.7%) of mucosal lymphomas. The intestinal margins were reported as free in 18 cases, but lymph nodes were affected in three of these cases.

**Figure 4:**
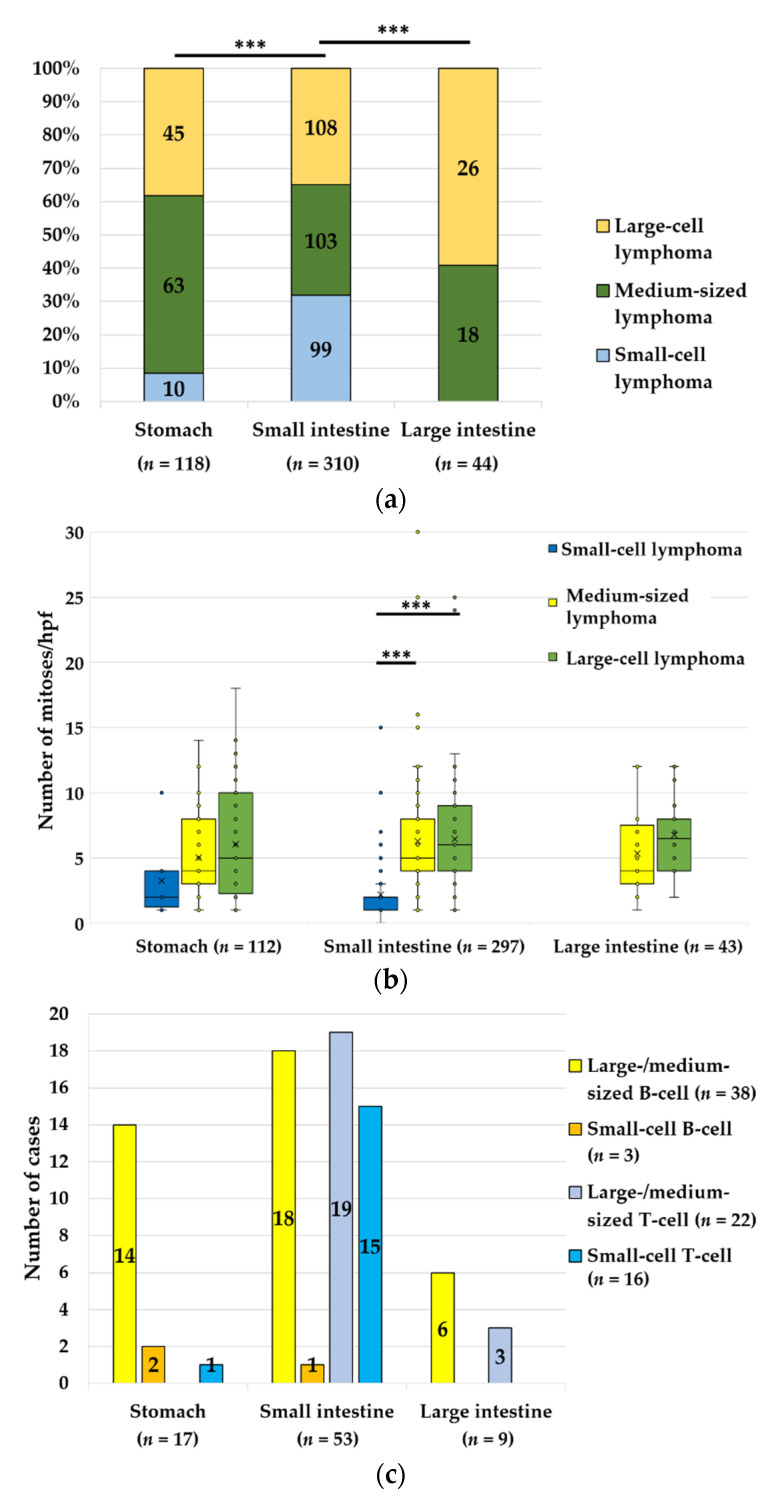

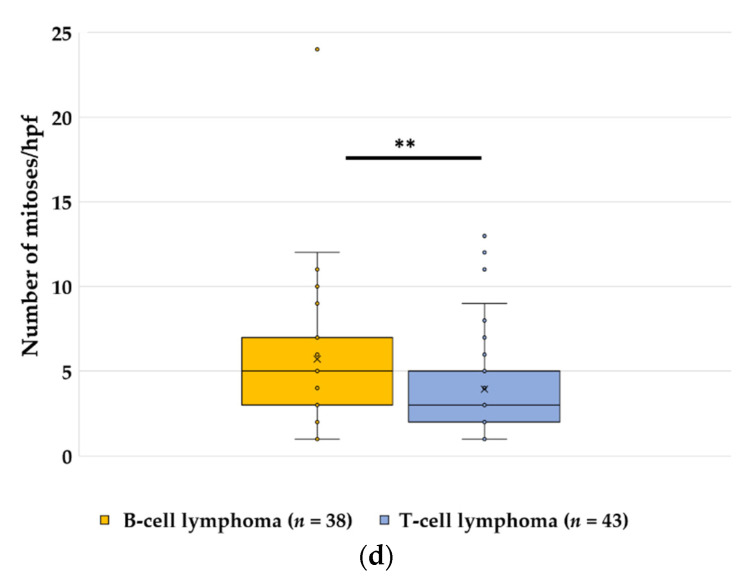
(**a**) Cell size of lymphomas in the stomach, small intestine and large intestine (*n* = 472). The cell size of lymphomas in the stomach and the small intestine (***, *p* < 0.001), as well as the small and the large intestine (***, *p* < 0.001), varied significantly. Small-cell lymphomas were frequently detected in the small intestine and absent in the large intestine. (NOS and lymphomas in more than one sample are not included in the graph.) (**b**) Distribution of feline lymphomas of varying cell sizes and their number of mitotic figures/hpf in feline lymphomas (*n* = 452) in different sites of the gastrointestinal tract. In the small intestine, the number of mitoses was significantly lower in small-cell lymphomas than in medium-sized (***, *p* < 0.001) and large-cell (***, *p* < 0.001) lymphomas. The wide range of mitotic numbers in all cell sizes of the alimentary lymphomas was noteworthy (intestine NOS and lymphomas in more than one sample are not included in the graph). (**c**) The distribution of feline lymphomas of varying cell sizes and immunophenotypes (*n* = 79) in different sites of the gastrointestinal tract shows that B-cell lymphomas were most common in the stomach and the large intestine, while T-cell lymphomas were most common in the small intestine. (NOS and lymphomas in more than one sample are not included in the graph.) (**d**) The number of mitotic figures/hpf was significantly higher in feline B-cell than in T-cell lymphomas (**, *p* = 0.008).

For further analyses, the detailed histomorphological findings of cell size (*n* = 472), average mitoses/hpf (*n* = 452) and immunophenotype (*n* = 91) were available in a varying number of cases depending on the tumour site, size and quality of the samples. In general, medium-sized and large-cell lymphomas were most common. The frequency of small-cell lymphomas varied significantly between the gastrointestinal locations (*p* < 0.001). Small-cell lymphomas were most common in the small intestine but were rare in the stomach and absent in the large intestine in the samples of our study (Figure 4a).

The number of mitoses varied from 0 to 30/hpf. In general, the mitotic count was significantly higher in intermediate-sized and large-cell lymphomas than in small-cell lymphomas of the small intestine (*p* < 0.001). Interestingly, there were 42 cases of small-cell lymphomas with >2 mitotic figures/hpf, including 11 cases with ≥5 mitotic figures/hpf. Furthermore, 45.5% of the intermediate-sized and 33.7% of the large-cell lymphomas had fewer than six mitotic figures/hpf (Figure 4b).

Immunohistochemical examination was performed in 91 cases as part of routine diagnostics (Figure 3b). Eight T-cell and four B-cell lymphomas were identified in intestine NOS cases, with lymphoma in the stomach and intestinal samples from the same animal. These 12 cases were not included in the following statistical analyses to avoid confusion, because the lymphomas affected multiple different locations in these cats, which would alter the case numbers. B-cell lymphomas were significantly more common in the stomach and the large intestine than in the small intestine (*p* < 0.001). In the small intestine, T-cell lymphomas were most frequent. Small-cell lymphomas were T-cell lymphomas significantly more often (87.5%) than medium-sized (*p* = 0.006) or large-cell (*p* < 0.001) lymphomas. The latter were B-cell lymphomas in 68.4% of cases. 

Figure 4c shows that the location and the size of neoplastic cells do not reflect the immunophenotype. Regarding the cell size, small-cell lymphomas were of T-cell origin significantly more often than medium-sized/large-cell lymphomas (*p* < 0.001). In general, the number of mitoses was significantly higher in B-cell lymphomas than in T-cell lymphomas (*p* = 0.008, Figure 4d).

### 3.3. Carcinoma, n = 122

Gastrointestinal carcinomas were most frequently found in older cats (≥10 years, 75.9%). The median age of the cats was 11 years (25% quartile: 10 years; 75% quartile: 14 years). In general, carcinomas of the intestine appeared more often in male castrated cats, but this was not statistically significant. In addition to 79 DSH, the most common breeds with gastrointestinal carcinoma were mixed breeds (*n* = 13), BSH (*n* = 7), Persian (*n* = 4) and Maine coon (*n* = 3) (Table 4).

Approximately two-thirds (58.1%) of the gastrointestinal carcinomas were located in the large intestine. About one-third of all carcinomas (32.0%) were found in the small intestine. Furthermore, in nine biopsies, the precise intestinal location could not be recognised, and information was not given in the clinical report. The maximum macroscopic size of gastrointestinal carcinomas which were resected as nodular masses (*n* = 26) ranged from 1.1 × 1.0 × 0.7 cm to 7.0 × 3.5 × 3.0 cm. 

**Figure 5:**
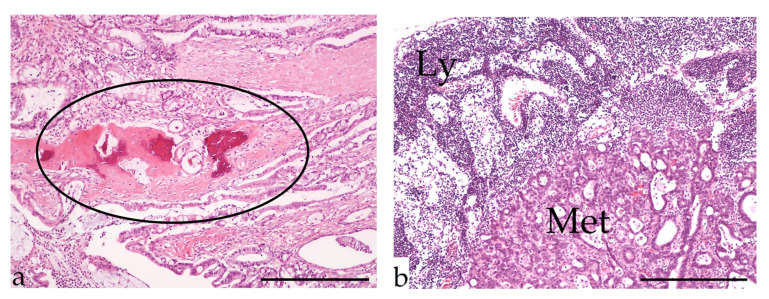
Intestinal carcinoma. (**a**) Osseous metaplasia (circle) in a tubular carcinoma of a 13-year-old male Chartreux; (HE stain, bar = 300 µm). (**b**): Metastasis of a tubular carcinoma (Met) in a regional lymph node (Ly) of a 16-year-old female spayed Persian; (HE stain, bar = 300 µm).

Histologically, gastrointestinal carcinomas appeared as poorly demarcated, non-encapsulated, highly infiltrative, epithelial neoplasms arising from the mucosa. Different growth patterns of tumour cells could be recognised, and in many cases, two or more of these growth patterns were found in one tumour. Most often (69.7%), a tubular/acinar growth pattern was dominant. Further cases were identified in which undifferentiated (19.7%) or mucinous (10.7%) tumour growth patterns dominated. Undifferentiated carcinomas often had a marked desmoplastic component, characterised by moderate to high amounts of fibrous stroma surrounding the neoplastic epithelial tubules. Metaplastic bone formation was present in five acinar carcinomas (Figure 5a). Papillary, signet-ring and (adeno)squamous differentiation were not observed as dominant patterns. Furthermore, no carcinoids were found. 

Regardless of the dominating growth pattern, the neoplastic cells were irregularly cuboidal to columnar with a moderate amount of eosinophilic cytoplasm with varying degrees of vacuolisation and goblet cell differentiation. There was moderate to marked anisokaryosis with aberrant nuclear shapes, occasional prominent nucleoli and 1–8 mitotic figures/hpf. The number of mitoses did not vary significantly between intestinal locations or growth patterns. In cases with a mucinous growth pattern, cystic spaces filled with mucus were present. There were single-cell necroses and mixed cellular inflammatory responses of varying degrees. Growth patterns did not differ significantly between the small and the large intestine (Table 4).

Transmural infiltrative growth of the carcinoma into the muscularis mucosa, submucosa, muscularis, serosa and adjacent mesenteric fat was common (86.9%). Surgical margins were free in 14 cases and affected in 77 samples (84.6%). In 31 samples, only pieces were submitted, and margins could not be evaluated. In carcinomas of the small intestine, the margins were more often free (20.7%) than in the large intestine (13.4%). However, this was not statistically significant. In 25 of 41 cases (61%) from which a regional lymph node was submitted, metastasis of the carcinoma was identified (Figure 5b). There was no significant difference between the occurrence of lymph node metastases in carcinomas of the small intestine (57.1%) and the large intestine (62.5%).

### 3.4. Spindle Cell Tumours, n = 29

Gastrointestinal spindle cell tumours were commonly diagnosed in older cats (≥10 years, 65.5%). The median age of the cats was 12 years (25% quartile: 8 years; 75% quartile: 13 years). There were three intact and 13 spayed females as well as 2 intact and 11 castrated male cats (Table 5). In addition to 22 DSH, 2 mixed breeds, 3 BSH, 1 Persian and 1 Cornish Rex were affected. Spindle cell tumours were mostly found in the intestine (small intestine *n* = 6, large intestine *n* = 10, intestine NOS *n* = 8) and less frequently found in the stomach (*n* = 5). Resected nodules (*n* = 8) had a size from 0.7 cm to 6.0 × 3.5 × 3.0 cm. 

**Figure 6:**
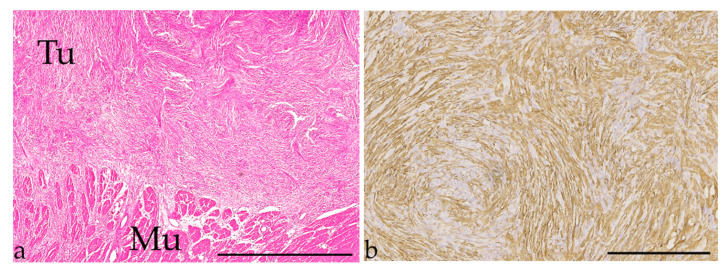
Intestinal leiomyosarcoma. (**a**): Histology of a spindle cell tumour (Tu) with invasive growth into the muscularis (Mu) of an 11-year-old male castrated DSH; (HE stain, bar = 2 mm). (**b**): Immunohistochemistry of the same tumour shows an intense expression of smooth-muscle alpha-actin in the neoplastic cells (leiomyosarcoma, IHC, bar = 300 µm).

Further differentiation was based on histopathological cell morphology and, in ten cases, also on immunohistochemistry. Eight leiomyosarcomas (Figure 6a,b), four haemangiosarcomas, one leiomyoma and one neurofibrosarcoma were identified. In 15 cases, malignant spindle cell tumours without further differentiation (NFD) were diagnosed, three of which had a very pleomorphic large-cell morphology. 

In general, spindle cell sarcomas consisted of a mass originating from mesenchymal cells. The cells were more or less spindle-shaped to polygonal, moderately to poorly differentiated and had a moderate amount of eosinophilic cytoplasm, a round to elongated nucleus finely stippled to the vesicular chromatin and one or more nucleoli. The anisocytosis and anisokaryosis were mild to moderate. The mitotic count ranged from 2 to 22 mitotic figures/10 hpf. All sarcomas were moderately inflamed with necrotic areas. Most of them (*n* = 21) were not completely excised. There were four cases where only parts of the mass were submitted and just four cases in which the complete mass was excised. Free margins were not correlated to the size or the subtype of the sarcoma. In two of the five submitted lymph nodes, sarcoma metastases were identified. Furthermore, liver metastases were submitted from one pleomorphic sarcoma.

### 3.5. Polyps, n = 23

**Figure 7:**
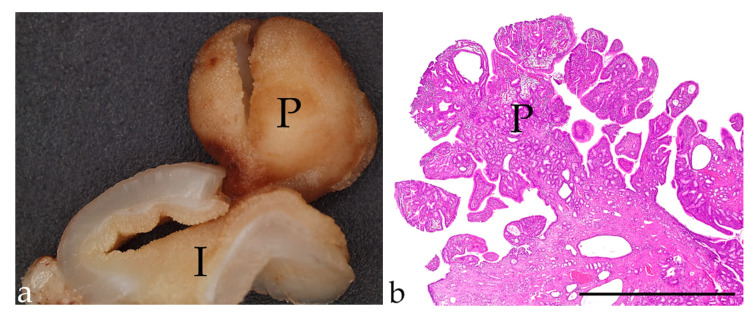
Intestinal polyp. (**a**): Macroscopy of a 1.5 cm polyp (P) within the small intestine (I) of a 13-year-old male castrated DSH. (**b**): Histology of a small intestinal polyp (P) in a 12-year-old female spayed DSH; (HE stain, bar = 2 mm).

Gastrointestinal polyps (Table 6) mostly occurred in older cats (≥10 years, 65.2%). The cats’ median age was 12 years (25% quartile: 8 years, 75% quartile: 14 years). There were one intact and seven spayed females as well as one intact and fourteen castrated male cats. In addition to 14 DSH, 3 mixed breeds, 2 Maine coon, 2 Chartreux, 1 BSH, and 1 Norwegian forest cat had gastrointestinal polyps (Figure 7a). They were mostly found in the intestine (small intestine *n* = 8, large intestine *n* = 12) and were less frequently found in the stomach (*n* = 3). Completely resected nodules (*n* = 5) had a size from 0.4 cm to 3.5 × 1.5 × 1.2 cm. 

Polyps were characterised by exophytic polypoid growth protruding from the mucosal surface. They were histopathologically formed of well-differentiated epithelium (Figure 7b). The cells were arranged in tubules and papillary projections supported by a fibrovascular stroma. Epithelial cells were cuboidal to columnar with a varying amount of eosinophilic to slightly basophilic cytoplasm. Occasionally, nuclei had lost their basal polarity. Single small nucleoli were present. There were mild anisocytosis and anisokaryosis with low mitotic activity. Varying degrees of inflammation could be seen. As polyp samples were often submitted fragmented, the margins could not be evaluated with certainty in most cases. However, in six cases, it was possible to confirm that polyps were completely resected.

### 3.6. Mast Cell Tumours, n = 7

All cats with gastrointestinal mast cell tumours were domestic short-haired cats with an age of 9–16 years (median age: 11 years, 25% quartile: 11 years, 75% quartile: 12 years): three spayed females and four castrated male cats. All mast cell tumours were located in the small intestine (Table 7). The maximum size of the mast cell tumours was 4.0 × 4.0 × 4.0 cm. 

**Figure 8:**
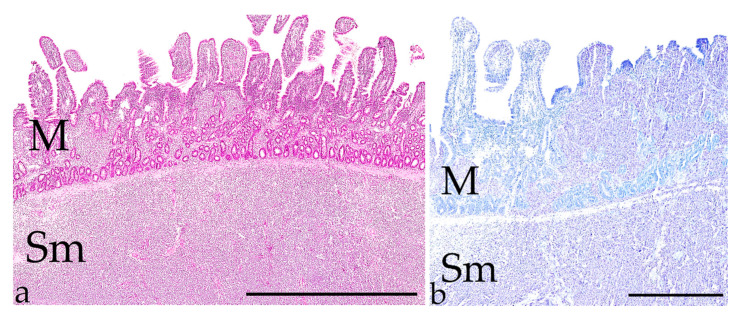
Intestinal MCT. (**a**)**:** Histology shows neoplastic infiltration of mast cells in the small intestinal mucosa (M) and submucosa (Sm) of a 13-year-old male castrated DSH; (HE stain, bar = 2 mm). (**b**)**:** Metachromatic staining of granules in neoplastic mast cells in the same tumour (Giemsa stain, bar = 600 µm).

The small intestinal wall was transmurally infiltrated by mast cells with fine or not visible granulation of the cytoplasm (Figure 8a). In cases of doubt, mast cell granules were identified by Giemsa staining (Figure 8b) or toluidine blue staining. Two mast cell tumours had a well-differentiated morphology, three were poorly (histiocytic) differentiated and two showed a pleomorphic morphology. One poorly differentiated mast cell tumour had a high amount of fibrous tissue, separating the mast cells into nests (sclerosing subtype). Single to multiple infiltrating eosinophils were present. Mitotic figures mostly ranged from 0–3/10 hpf but were 10/hpf in one pleomorphic mast cell tumour. 

Further immunohistochemical examination revealed diffuse intracytoplasmic expression of the cKit receptor in all cases. Additionally, some mast cells with a stippled perinuclear expression pattern were observed in three cases. 

The margins were free in only one case of a moderately differentiated mast cell tumour. The lymph nodes were submitted in three cases (2× well differentiated, 1× pleomorphic mast cell tumour), and all of them contained numerous mast cells classified as HN2 or HN3 according to Weishaar et al., 2014 [46].

### 3.7. miRNA Analysis 

**Figure 9:**
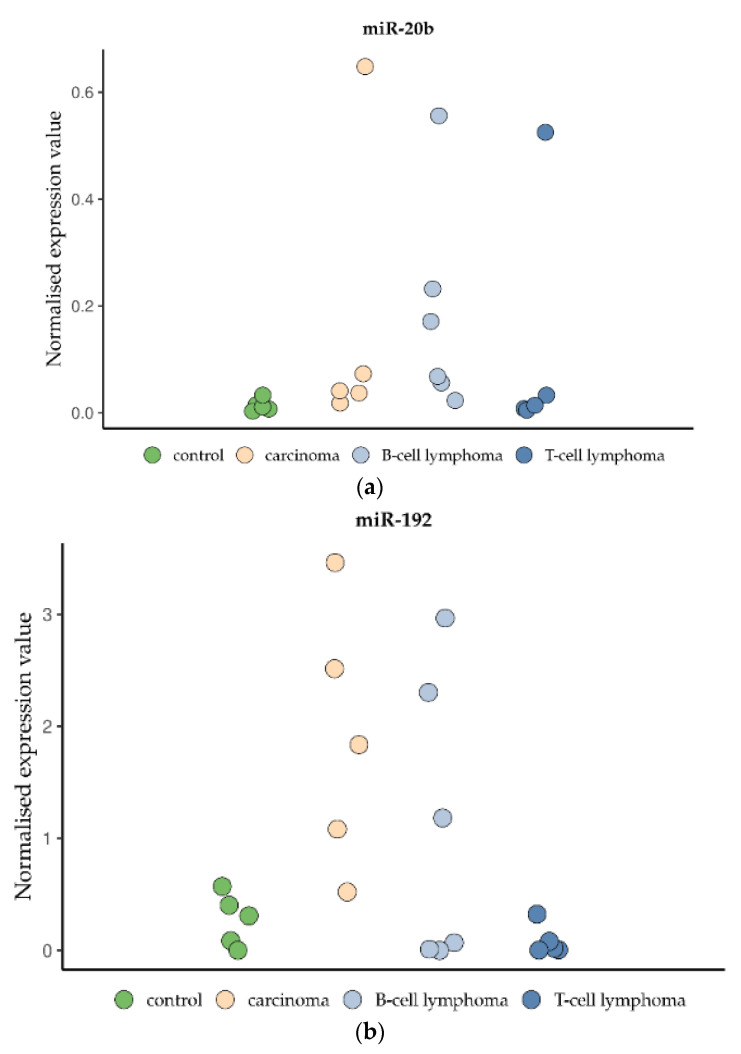
Expression levels of miR-20b (**a**) and miR-192 (**b**) in samples of small intestinal tubular carcinomas (*n* = 5), large-cell B-cell lymphomas (*n* = 6) and small-cell T-cell lymphomas (*n* = 5) versus normal intestinal control tissue (*n* = 5). Absolute quantification was carried out by ddPCR. The absolute miRNA copy number was related to the internal normaliser RNU6B. Results are shown as normalised expression values.

The expression of two miRNAs (probably pro-oncogenic miR-20b and probably tumour-suppressive miR-192) were tested in five randomly chosen carcinomas (5× tubular), six large-cell B-cell lymphomas (3–14 mitoses/hpf) and five small-cell T-cell lymphomas (1–4 mitoses/hpf) of the small intestine and were compared to five controls without inflammation or a tumour (Table 2). Absolute quantification was carried out by ddPCR. The absolute miRNA copy number was related to the internal normaliser RNU6B. These normalised expression values are shown in Figure 9. Due to the small number of samples and the ranges of values, we decided to refrain from calculating statistics to avoid non-reliable results.

The expression of miR-20b in the controls was slightly lower (range 0.003–0.03 miR-20b/U6) than that in carcinomas (range 0.02–0.65 miR-20b/U6), B-cell lymphomas (range 0.02–0.56 miR-20b/U6) and T-cell lymphomas (range 0.005–0.53 miR-20b/U6) (Figure 9a). In each tumour group, one animal showed strikingly high expression values. The increased expression of miR-20b was highest in a tubular carcinoma of an 8-year-old Russian blue (60× fold), a B-cell lymphoma with 5 mitoses/hpf of an 11-year-old DSH (50× fold) and a T-cell lymphoma with 1 mitosis/hpf of a 7-year-old DSH (50× fold). 

The expression of miR-192 in the controls (range 0.001–0.57 miR-192/U6), was lower than that in carcinomas (range 0.52–3.46 miR-192/U6) and B-cell lymphomas (range 0.0001–2.97 miR-192/U6) (Figure 9b). In contrast, there was no difference in miR-192 between the T-cell lymphoma (range 0.002–0.32 miR-192/U6) and control group. The miR-192 values of carcinomas and B-cell lymphomas were distributed evenly within their ranges. Interestingly, the two cats with the highest miR-20b values in carcinoma and B-cell lymphoma also had the highest miR-192 values. In contrast, the cat with the highest miR-20b value (0.53 miR-20b/U6) in a T-cell lymphoma with 1 mitosis/hpf had a very low miR-192 value (0.005 miR-192/U6). In general, no correlation of the mitotic count in lymphomas and miRNA values (miR-20b and -192) has been obvious so far.

## 4. Discussion

In the present study, gastrointestinal non-inflammatory masses from 860 cats were analysed retrospectively from routine pathology reports. Similar to other studies [4,47], predominantly malignant neoplasms were found in the gastrointestinal tract of cats. Although polyps are classified by WHO classification as a non-neoplastic tumour-like lesion [39], polyps were included in the present study because they are an important (benign) differential diagnosis in cases with gastrointestinal masses [6,40,41]. No data about clinical history, treatment, survival time or further affected organs were available in our study.

A breed predisposition for alimentary neoplasms was not identified in the cases of our cohort, as about 80% of the cats were domestic short-haired cats or mixed breeds. In other publications, mainly from the United States, Siamese cats were overrepresented [7,47], but in our cohort, which came from Germany and European countries, Siamese cats were rare (*n* = 10). 

In the present study, the high proportion of gastrointestinal lymphomas (79.0%) was surprising. Older studies found lower proportions (41% to 52.3%), but they evaluated variable parts of the alimentary tract [4,7,48]. Improved diagnostics and therapeutic options, as well as an increasing willingness of patient owners to perform more advanced diagnostic measures, such as the immunohistochemical subtyping of lymphomas, may have contributed to the higher proportion of gastrointestinal lymphomas in the present pathology-based investigation. The low percentage of spindle cell tumours and mast cell tumours, along with the low incidence of neoplasms in cats younger than seven years of age, were consistent with the observations of other authors [7,47,48,49]. However, we should be aware that data from pathological cases do not reflect the proportion of tumour diseases in cats in daily veterinary practice, since endoscopic or laparoscopic sampling is often only performed in the further course of the diagnosis of tumour diseases and only if advanced treatment is planned.

Alimentary lymphomas were most frequently found in the small intestine in this study, as previously described by other authors [7,19,50]. In general, there is a lot of terminological confusion as a result of mixing cytological and clinical nomenclature with histopathological and immunohistochemical findings [22]. Avallone et al. pointed out that the term “grade” in lymphomas is commonly and incorrectly applied to indicate the expected clinical course of the untreated disease (e.g., high-grade B-cell lymphoma) [20]. Thus, the authors highly recommend using the terms indolent, intermediate and aggressive behaviour to stratify lymphomas by their predicted clinical course (when untreated) and to divide histological grades into low-, medium- and high-grade categories according to mitotic activity [20]. A limitation of our study was the fact that not all cases were available for statistical analysis, as the precise gastrointestinal location, mitotic count, cell size or immunophenotype were not always reported for different reasons (incomplete reports, quality and size of the samples). However, because of the high number of cases in this study, the results are considered as valid. 

Lymphomas of the lymph nodes can be classified as low-grade if they have fewer than five mitotic figures/hpf, medium-grade if they have between 5 and 10 and high-grade if they have over 10 mitotic figures/hpf [26]. Based on studies that differentiate intestinal lymphomas from inflammatory bowel disease, 0–2 mitotic figures have been described in lymphomas [25]. Interestingly, there were several cases of alimentary small-cell lymphomas with mitotic numbers of >2 mitotic figures/hpf (*n* = 42) in our study (including 11 cases with ≥5 mitotic figures/hpf). Furthermore, 45.5% of the intermediate-sized and 33.7% of the large-cell lymphomas had fewer than six mitotic figures. This shows that the mitotic count must be considered with caution as a prognostic factor until valid studies with relevant case numbers have been performed. In our cases, lymphomas with 0 mitoses occurred; however, they were mainly transmural masses or markedly diffuse neoplastic infiltrates, and inflammatory bowel disease was excluded with certainty. Furthermore, doubtful cases had been excluded (see Materials and Methods).

Immunohistochemical differentiation was performed on 91 lymphomas in the present study, which is the highest number of cases in a study published so far. As mentioned above, only B-cell lymphomas were found in the stomach, whereas T-cell lymphomas predominantly appeared in the small intestine [50,51,52]. B-cell lymphomas occurred in the small and the large intestine in the present study, as has been previously reported [50]. 

As described in the literature [1,53], intestinal small-cell lymphomas were T-cell lymphomas in almost all cases of the present study as well. Small-cell T-cell lymphomas with few mitoses (<2 mitosis/hpf) are said to have a better prognosis than other alimentary lymphomas, but they are much more difficult to distinguish from inflammatory bowel disease. This is probably the reason why many studies deal with this particular form of alimentary lymphoma [25,53,54,55,56]. However, it should be noted that there were several cases with higher mitotic counts (2–10/hpf) in small-cell lymphomas in our study, but unfortunately, no prognostic or therapeutic data were available.

With the present study on numerous feline alimentary lymphomas, characterised in detail, it becomes clear once again that there are several cases that do not fit into this very narrow two-type system of indolent small-cell, mitotic count <2 T-cell lymphomas and intermediate or aggressive medium-, mitotic count >2 large-cell B-cell lymphomas. For example, there were three small-cell B-cell lymphomas (two in the stomach and one in the small intestine) in this study. T-cell lymphomas appeared as medium- or large-sized lymphomas, and, of our total T-cell cases, they predominated, which has also been previously described in the literature [57]. 

In contrast to other investigations [22,50], we had no cases of lymphomas which were negative for T- and B-cell markers or which were described as T-cell-rich B-cell lymphomas. This may be due to the fact that we only included certain cases in this retrospective study. Thus, cases with such special patterns may have been interpreted with restrictions and would have led to a precautionary exclusion from our study, which was based solely on pathology reports with a definitive diagnosis. 

In conclusion, the results of the present study show that, especially due to the lack of a prognostically relevant grading system for feline alimentary lymphomas, a detailed characterisation, including tumour cell size, mitotic count and immunological phenotype, is necessary for the individual workup of the patient. Further controlled studies are needed to reliably evaluate cases with less typical patterns of findings regarding their clinical, prognostic and therapeutic significance.

The proportion of carcinomas (*n* = 122) in the present study (14.2%) was similar to that of other studies [4]. Consistent with their findings [7,48,58], carcinomas occurred much more frequently in the large intestine (58.1%) than in the small intestine (32.0%) and the stomach (2.5%). Contrary to this, some studies found carcinomas mainly in the small intestine [22,47,59]. In our cohort, carcinomas of the intestine were most common in male cats (about 70%). This was also reported in intestinal carcinomas in another study [58]. As mentioned before, gastric carcinomas in cats were very rare [1,60]. 

Histologically, different growth patterns could be recognised in gastrointestinal carcinomas, but there is no prognostic relevance of these growth patterns known in veterinary medicine [22]. In the current study, tubular gastric and acinar intestinal carcinomas were the most common type, accounting for approximately 69.7% of the gastrointestinal carcinomas, similar to the numbers described in the literature [47,58]. However, two or more different growth patterns often appeared within one carcinoma. We identified osseous metaplasia, which could be conspicuous on imaging, in five carcinomas. Yet, osseous metaplasia was also reported in leiomyosarcoma [38]. 

In this study, spindle cell tumours were equally common in the small and the large intestine. Other authors, in contrast, found more spindle cell tumours in the small intestine than in the large intestine [7]. Some of the sarcomas in our study were characterised in more detail by immunohistochemistry if the client was interested. This enabled the reliable diagnosis of eight leiomyosarcomas and one neurofibrosarcoma. Four haemangiosarcomas were easily identified by histomorphological criteria. Individual studies of gastrointestinal haemangiosarcomas [34,35] as well as of intestinal [36,37,38] and gastric [37] leiomyosarcomas have been described in cats, and the findings mainly correspond to the results of the present study. Gastrointestinal stromal tumours were not identified in our cohort.

In the literature, polyps were mostly described in the feline stomach or small intestine [6,40,41,61,62]. In contrast, in our study, polyps of the large intestine were found in 12 of 23 cases. It was interesting to see that there were no obvious signs of malignant transformation into carcinoma. The ages of the affected cats with polyps of the large intestine and of those with carcinomas of the large intestine were in a similar range. Epithelial proliferations of the large intestinal mucosa were benign in 12 of 84 cases (14.3%), and surgical removal may be curative and can provide the basis for histological diagnosis. However, we do not have any information about the clinical outcome, and it cannot be excluded that there were malignant areas in other sites.

Feline mast cell tumours (*n* = 7) were rare in the present study (0.8%), which is consistent with the literature [7]. Mast cell tumours of the large intestine were not present here but have been described by other authors [48]. The diagnosis of gastrointestinal mast cell tumours here and in other studies was largely only possible through the use of special stains and/or immunohistochemistry [22]. Sclerosis was seen in one poorly differentiated mast cell tumour in our study, but higher numbers (*n* = 50) were previously described in detail [31]. Barrett et al. studied 31 cats with gastrointestinal mast cell tumours and found several other affected organs such as lymph nodes, the liver and the spleen [32]. The submitted lymph nodes were infiltrated by mast cells in three of three cases in our study, but we had no information about other affected organs. Prognosis in cases of gastrointestinal mast cell tumours seems to be variable, and a combination of surgical and medical treatment may be useful [31,32]. Interestingly, concurrent intestinal mast cell tumours and small T-cell lymphoma have been described in some cases [33], which may influence the outcome. The low mitotic count in our study was in line with the literature [30]. All of our mast cell tumours had a diffuse intracytoplasmic cKit expression. Contrary to this, other authors found cKit expression in only about one-third of the cases [30,63]. Again, this may be caused by the exclusion of uncertain cases in our study.

It has been shown that intestinal neoplasms in cats (except for lymphomas) should be resected with 4 cm margins in oral and aboral directions [49]. In our study, large intestinal carcinomas especially lacked free margins, probably due to the anatomical limitations of surgery. Additional samples of regional lymph nodes were submitted in 307 cases of intestinal neoplasms, and 68% of them were affected by metastases, as reported by other studies [47,48,49]. Metastases were identified as a negative prognostic factor [48]. Thus, if lymph node tissue and other organs (liver, spleen, peritoneum) are not sampled and examined histologically, the clinical staging or further prognostic assessment of the individual case is hampered [19].

In search for less invasive diagnostic tools, this study investigated molecular biological methods and now provides the first report on the expression of two selected miRNAs (miR-20b and miR-192) in feline intestinal cancer. These two miRNAs were chosen, as they have been described as being mis-regulated in intestinal T-cell lymphoma in dogs with the highest fold change [18]. Additionally, TaqMan miRNA assays were available and yielded reliable results. In this pilot study, we compared miRNA values from a limited number of carcinomas and B- and T-cell lymphomas with control samples from normal small-intestinal tissue. In order to avoid miRNA shifts due to different intestinal locations, all samples were chosen from the small intestine. We were able to show that ddPCR using TaqMan miRNA assays is a robust technique for quantifying miRNA in feline samples, as the triplicates showed high reproducibility. However, the quantification of miRNA by ddPCR is a relatively new field, and no commonly accepted procedure for normalisation has been published. Using RNU6B is the most common approach in quantitative polymerase chain reaction (qPCR) and ddPCR [18,64], but it is under debate [65]. 

In our study, miR-20b was found to be slightly up-regulated in all tumour samples with one carcinoma, three B-cell lymphomas and one T-cell lymphoma showing a bigger shift. The miR-20b is part of the miR-106a~363 polycistronic cluster, which is correlated with pro-oncogenic effects in different tumours including T-cell leukaemia in humans [66] and T-cell lymphoma in dogs [18]. Our results in B- and T-cell lymphomas are in accordance with these studies, emphasising the critical role of miR-20b and the whole complex in carcinogenesis across species [67]. It was interesting to note that miR-20b was also up-regulated in intestinal carcinomas in cats. Comparative analyses are now necessary to relate these results to inflammatory lesions of the feline intestine to evaluate the use of miR-20b as a diagnostic biomarker.

The miR-192 is part of the miR-194-2~miR-192 and miR-194-1~miR-215 clusters, which have been described as having tumour-suppressive functions and therefore as being down-regulated in several human cancer types [68] as well as in canine intestinal T-cell lymphoma [18]. On the other hand, miR-192 has been described as being up-regulated in gastric cancer [69] and small-cell lung cancer in humans [64,70]. In our study, miR-192 was up-regulated in all carcinomas and in three of six B-cell lymphomas, but not in small-cell mitotic count <2 T-cell lymphoma samples. The reason for these differences needs more in-depth analysis, but the fact that these were small-cell lymphomas with a low mitotic count may play a role. Further analysis of inflammatory bowel disease is necessary to evaluate this hypothesis. 

These differences, together with the opposing expression shifts in human cancer types [70], show that more research is needed to resolve the miRNA profiles of healthy tissue and different cancer types to obtain more information on cancer characterisation in different tissues and various species. For further studies, the case numbers and miRNAs examined have to be increased. Several other miRNAs have been described as being mis-regulated in canine lymphomas [18,71,72,73] and as possibly being mis-regulated in cats as well. Furthermore, another approach, such as next generation sequencing (NGS) or microarrays, may be used to screen for relevant miRNAs, as little is known about the miRNA profiles of feline healthy and cancer tissues [16,17]. Additionally, analyses of serum samples are needed to prove the up-regulation of miR-20b and miR-192 and possibly other miRNAs detected in this sample type. Serum samples could be used for liquid biopsy, which is a promising field for new diagnostic possibilities that are reliable, time-saving and non-invasive [15].

## 5. Conclusions

This study shows, in a high case number, that neoplasms of the gastrointestinal tract are mostly malignant in cats. In many cases, lymph nodes were involved, and there was a lack of free margins. Lymphomas were the most common neoplasms in the feline gastrointestinal tract, followed by carcinomas, sarcomas and mast cell tumours. It should be noted that, apart from the typical small-cell, mitotic count <2 T-cell lymphomas and large-cell B-cell lymphomas with high mitotic numbers, there are several variants that have not been classified so far, and further studies are needed. 

In the search for a new diagnostic tool, the data of a pilot study on miRNA concentrations in feline small intestinal neoplasms revealed promising results. Especially, the diagnostic purpose of miR-20b and miR-192 as potential biomarkers for intestinal cancer should be further evaluated.

## Figures and Tables

**Table 1:** Primary antibodies and pre-treatment used in routine immunohistochemistry in this study.

Antigen (Clone)	Species	Supplier	Dilution	Pre-Treatment
CD3 (F7.2.38)	mouse	Dako ^1^ #M7254	1:100	EDTA buffer
CD79a (HM57)	mouse	BioRad ^2^ #MCA2538GA	1:3000	EDTA buffer
CD20	rabbit	Epria ^3^ #RB-9013-P1	1:100	EDTA buffer
cKit/CD117	rabbit	Dako ^1^ #A4502	1:150	EDTA buffer
Smooth muscle Alpha actin (1A4)	mouse	Dako ^1^ #M0851	1:100	target retrieval solution buffer
Glial fibrillary protein	rabbit	Dako ^1^ #Z0334	1:300	EDTA buffer

^1^ Dako Denmark A/S, Glostrup, Denmark; ^2^ Bio-Rad Laboratories Inc., Hercules, CA, USA; ^3^ Epria Netherlands B.V., Breda, Netherlands.

**Table 2:** Signalment of the cats included in the pilot study of miRNA analysis (*n* = 21).

Group	Breed	Age (Years)	Sex
B-cell lymphoma (*n* = 6)	4 DSH, 1 mix, 1 Somali	4–16	1 m, 1 mc, 1 f, 3 fs
T-cell lymphoma (*n* = 5)	5 DSH	6–14	3 mc, 2 fs
Carcinoma (*n* = 5)	4 DSH, 1 Russ. blue	8–14	1 m, 4 mc
Controls (*n* = 5)	3 DSH, 1 BSH, 1 Russ. blue	1–13	2 m, 3 mc

Legend: BSH = British shorthair; DSH = domestic short-haired cat; f = female; fs = female spayed; m = male; mc = male castrated; mix = mixed breed; Russ. blue = Russian blue.

**Table 3:** Signalment of the cats with gastrointestinal lymphomas in the present study (*n* = 679).

Site	Breed	Age (Years)	Sex
Stomach (*n* = 118)	74 DSH, 8 Maine coon, 7 BSH, 6 mix, 3 Chartreux, 3 Persian, 3 Russ. blue, 3 Siamese, 3 Thai, 2 Burmese, 2 Norw. forest cat, 4 others	1–17 median: 10	8 m, 48 mc, 10 f, 52 fs
Small intestine (*n* = 310)	233 DSH, 27 mix, 9 Maine coon, 9 Norw. forest cat, 6 Persian, 4 BSH, 4 Siamese, 4 Turkish Angora, 3 Bengal, 3 ELH, 2 ASH, 6 others	1–18 median: 11	22 m, 157 mc, 23 f, 108 fs
Large intestine (*n* = 44)	32 DSH, 4 BSH, 3 mix, 2 Maine coon, 3 others	1–15 median: 10	2 m, 24 mc, 2 f, 16 fs
Intestine NOS (*n* = 182)	123 DSH, 20 mix, 11 BSH, 5 Maine coon, 3 ELH, 2 Bengal, 2 DLH, 2 Siamese, 14 others	1–16 median: 10	27 m, 76 mc, 18 f, 61 fs
Intestine and stomach (*n* = 25)	15 DSH, 2 BSH, 2 mix, 6 others	4–14 median: 11	1 m, 10 mc, 1 f, 13 fs

Legend: ASH = American shorthair; BSH = British shorthair; DLH = domestic long-haired cat; DSH = domestic short-haired cat; ELH = exotic long-haired cat; f = female; fs = female spayed; m = male; mc = male castrated; mix = mixed breed; Norw. forest cat = Norwegian forest cat; NOS = not otherwise specified; Russ. blue = Russian blue.

**Table 4:** Signalment of the cats with gastrointestinal carcinoma in the present study (*n* = 122).

Site	Breed	Age (Years)	Sex	Morphology
Stomach (*n* = 3)	1 Bengal, 1 Norw. forest cat, 1 Siberian	9–12 median: 10	1 mc, 2 fs	2 tubular, 1 undiff.
Small intestine (*n* = 39)	26 DSH, 5 mix, 1 Balinese, 1 Bengal, 1 Birman, 1 BSH, 1 ELH, 1 Oriental, 1 Russ. blue, 1 Siamese	5–19 median: 11	7 m, 20 mc, 3 f, 9 fs	25 acinar (2× bone metaplasia), 4 mucinous, 10 undiff.
Large intestine (*n* = 71)	47 DSH, 8 mix, 6 BSH, 3 Maine coon, 3 Persian, 2 Chartreux, 1 BLH,1 Norw. forest cat	3–23 median: 11	5 m, 33 mc, 12 f, 21 fs	54 acinar (3× bone metaplasia), 9 undiff., 8 mucinous
Intestine NOS (*n* = 9)	6 DSH, 1 Balinese, 1 Persian, 1 Sphynx	7–17 median: 12	6 mc, 3 fs	5 acinar, 3 undiff., 1 mucinous

Legend: BLH = British longhair; BSH = British shorthair; DSH = domestic short-haired cat; ELH = exotic long-haired cat; f = female; fs = female spayed; m = male; mc = male castrated; mix = mixed breed; Norw. forest cat = Norwegian forest cat; NOS = not otherwise specified; Russ. blue = Russian blue; undiff. = undifferentiated.

**Table 5:** Spindle cell tumours of the feline gastrointestinal tract in the present study (*n* = 29).

Tumour	Site	Breed	Age (Years)	Sex
Leiomyosarcoma (*n* = 8)	5 small intestine, 1 large intestine, 1 stomach, 1 intestine NOS	7 DSH, 1 Cornish Rex	2–15	4 mc, 4 fs
Haemangiosarcoma (*n* = 4)	2 large intestine 2 intestine NOS	3 DSH, 1 Persian	5–10	1 mc, 3 fs
Leiomyoma (*n* = 1)	small intestine	DSH	13	fs
Neurofibrosarcoma (*n* = 1)	large intestine	DSH	7	fs
Spindle cell sarcoma NFD (*n* = 12)	6 large intestine, 3 small intestine, 3 stomach	7 DSH, 3 BSH, 2 mix	5–16	2 m, 5 mc, 3 f, 2 fs
Pleomorphic sarcoma NOS (*n* = 3)	3 intestine NOS	3 DSH	12–18	1 mc, 1 f, 1 fs

Legend: BSH = British shorthair; DSH = domestic short-haired cat; f = female; fs = female spayed; m = male; mc = male castrated; mix = mixed breed; NFD = not further differentiated; NOS = not otherwise specified.

**Table 6:** Polyps of the feline gastrointestinal tract in the present study (*n* = 23).

Site	Breed	Age (Years)	Sex
Stomach (*n* = 3)	1 BSH, 1 DSH, 1 mix	7–14	1 mc, 2 fs
Small intestine (*n* = 8)	8 DSH	11–16	1 m, 5 mc, 2 fs
Large intestine (*n* = 12)	5 DSH, 2 Chartreux, 2 Maine coon, 2 mix, 1 Norw. forest cat	1–15	8 mc, 1 f, 3 fs

Legend: BSH = British shorthair; DSH = domestic short-haired cat; f = female; fs = female spayed; m = male; mc = male castrated; mix = mixed breed; Norw. forest cat = Norwegian forest cat.

**Table 7:** Feline intestinal mast cell tumours included in the present study (*n* = 7, all DSH).

Case No	Sex	Age	Cellular Differentiation	Mitotic Figures/hpf	cKit Expression Pattern
1	mc	11	moderate	3	diffuse
2	fs	11	good	1	diffuse + stippled
3	fs	13	sclerosing	1	diffuse
4	fs	11	good	1	diffuse + stippled
5	mc	10	pleomorphic	10	diffuse
6	mc	9	pleomorphic	2	diffuse
7	mc	16	poor (histiocytic)	1	diffuse + stippled

Legend: DSH = domestic short-haired cat; fs = female spayed; mc = male castrated.

## Data Availability

The raw data of the results presented in this study are available on request from the corresponding author.

## Abbreviations

ASH = American shorthair; BLH = British longhair; BSH = British shorthair; ddPCR = droplet digital polymerase chain reaction; DLBCL = diffuse large B-cell lymphoma; DLH = domestic long-haired cat; DSH = domestic short-haired cat; EATL = enteropathy-associated T-cell lymphoma; ELH = exotic long-haired cat; f = female; fs = female spayed; hpf = high power field; LGL = large granular lymphoma; m = male; mc = male castrated; miR = miRNA; mix = mixed breed; MALT = mucosa-associated lymphatic tissue; NGS = next generation sequencing; Norw. forest cat = Norwegian forest cat; NOS = not otherwise specified; qPCR = quantitative polymerase chain reaction; Russ. blue = Russian blue; undiff. = undifferentiated; WHO = World Health Organisation.
